# Posttraumatic Stress Symptoms and Anger Among Police Officers Following a Fatal Knife Attack on a Team Member

**DOI:** 10.3390/healthcare14030295

**Published:** 2026-01-24

**Authors:** Anna Koch-Scharwatt, Ulrich Wesemann

**Affiliations:** 1 Department of Psychiatry, Psychotherapy and Psychotraumatology, Bundeswehr Hospital Berlin, Scharnhorststr. 13, 10115 Berlin, Germany; 2 Clinic for Psychiatry and Psychotherapy, Charité-Universitätsmedizin Berlin, Campus Charité Mitte, Charitéplatz 1, 10117 Berlin, Germany

**Keywords:** anger, knife attack, mental health, police officers, PTSD, risk factors

## Abstract

Mental disorders and impairments are part of the occupational risk for emergency personnel. This study examines the impact of a deadly knife attack on police officers’ mental health. **Aims**: We hypothesized that police officers who knew the deceased team member would report higher levels of psychological distress compared to those who did not, regardless of the deployment status. **Methods**: Six months after a fatal knife attack in which a police officer was killed, a total of N = 245 officers participated in the study. Of these, n = 115 reported knowing the victim personally, n = 126 did not (n = 78 deployed; n = 176 not deployed), while n = five did not provide any information. Posttraumatic stress disorder (PTSD), posttraumatic stress symptoms (PTSS), anger and adverse childhood experiences (ACEs) were assessed using questionnaires. Chi-square tests examined group differences in probable PTSD prevalence; *t*-tests assessed differences in anger and Posttraumatic Stress Disorder Checklist for DSM-5 (Diagnostic and Statistical Manual of Mental Disorders, Fifth Edition; DSM-5; PCL-5) symptom scores. Linear regression analyses tested deployment, acquaintance with the victim, gender, and childhood emotional neglect as predictors. **Results**: Police officers who personally knew the deceased colleague exhibited significantly higher PTSS scores. In addition, the deployed group showed significantly higher trait anger than the non-deployed. Acquaintance with the victim and emotional neglect in childhood were significantly related to negative cognitions, whereas deployment to the knife attack or gender were not. **Discussion**: Police officers with a personal connection to the deceased showed significantly higher mental health impact than those with direct exposure alone, placing them in a higher-risk group due to increased exposure to feelings of guilt and shame due to their professional role. Police officers who were emotionally neglected in their childhood may be more prone to negative cognitions in adulthood, when faced with critical events. These results underline the importance of addressing risk factors in both pre-deployment training and post-event debriefing, especially with regard to anger management after major critical incidents.

## 1. Introduction

Occupational trauma among emergency responders such as police officers represents a distinct form of psychological strain, combining acute exposure to violence with moral, ethical, and relational challenges that exceed standard typical presentation of a trauma [1,2]. However, despite extensive research on occupational trauma, existing studies have rarely examined how situational exposure to a critical incident and relational proximity to the victim jointly contribute to posttraumatic outcomes among police officers, representing a specific gap addressed by the present study. Police officers frequently encounter critical incidents such as death, threats and serious injury, with cumulative exposure increasing vulnerability to posttraumatic stress symptoms (PTSS, referring to dimensional symptom severity rather than a clinical diagnosis), depression, and maladaptive anger regulation [3,4,5]. Prior research indicates a prevalence of posttraumatic stress disorder (PTSD, defined as a categorical diagnosis according to the International Classification of Diseases, 11th Revision; ICD-11) of roughly 10% among first responders [6,7]. Recent studies show that the prevalence of PTSD among police personnel varies from 4.1% to 13.9% [8].

Anger, as a fundamental emotional response, is increasingly recognized as both a symptom and a sustaining factor of PTSD [9,10]. Research indicates that heightened anger reactivity in first responders might result from perceived injustice, loss of control, or moral transgression associated with traumatic exposure, and this occurrence appears to be more prevalent among first responders than in civilian populations [11]. Police officers appear to be even more vulnerable [12]. Anger can increase the risk of cardiovascular disease and other comorbid illnesses, and it is strongly associated with the prevalence and severity of PTSD [13,14]. Chronic anger correlates with avoidance behavior, interpersonal conflict, and weaker treatment outcomes in populations exposed to trauma [15,16], highlighting anger as a clinically relevant factor rather than a secondary emotional reaction.

While direct trauma exposure, such as being deployed to a critical incident, is a known risk factor [17], exposure to behaviors that go against deeply held moral beliefs has also been demonstrated to contribute to the development and maintenance of PTSD [18]. According to Murray et al. being familiar with the deceased increases emotional attachment and deepens mourning reactions, which increases vulnerability to PTSD [19]. Despite this evidence, the combined effects of deployment status and personal familiarity with the deceased have not been systematically investigated in police populations following fatal incidents.

Developmental vulnerabilities, such as the consequences of childhood maltreatment, represent an additional and frequently overlooked element influencing stress reactivity and cognitive processing of trauma [20,21]. Childhood emotional neglect disrupts affect regulation, attachment security, and self-concept formation, which in turn moderates later trauma responses [22,23,24]. Although temporally distal, childhood emotional neglect may increase vulnerability to trauma-related negative cognitions and emotion dysregulation when police officers are confronted with incidents involving loss or perceived failure. According to the cognitive model of PTSD [25], early experiences can predispose individuals to form enduring negative beliefs about themselves and their environment (e.g., guilt, shame, or mistrust), especially when faced with trauma related to loss or perceived failure [25,26].

Taken together, occupational exposure, relational proximity to the deceased, and developmental vulnerability can be understood within an integrative cognitive–developmental framework, in which prior adverse experiences shape cognitive appraisals and emotional responses to duty-related traumatic events.

It is essential to comprehend these interrelations for the formulation of trauma-informed therapies and peer-support initiatives specifically designed for police culture and hierarchy, while addressing both individual susceptibility and organizational accountability [27]. This research is a component of the CASH (Calamities, Anxiety, Stress, Hostility)-Force project, which is designed to identify mental health burdens among emergency personnel across a broad range of major incidents, including both human-made events and natural disasters, and to guide post-incident intervention strategies [4]. Within this broader framework, the present study focuses specifically on police officers following a fatal, human-caused duty-related incident [4].

This study investigates the effects of deployment status, familiarity with the deceased, and anger expression patterns among police officers after a fatal police incident, thereby integrating occupational exposure and relational proximity, considering the interplay of occupational exposure, relational proximity, developmental adversity, and childhood emotional neglect on PTSS.

We therefore hypothesized that police officers who personally knew the fatally injured colleague were more likely to have higher levels of PTSS, regardless of their deployment status during the critical incident. Furthermore, we hypothesized that officers who were deployed to the scene will demonstrate higher trait anger. Finally, we hypothesized that childhood emotional neglect and acquaintance with the deceased colleague will significantly predict negative cognitions as a core cognitive symptom dimension of PTSD.

## 2. Methods

### 2.1. Survey Strategy

Data were collected through a written survey administered six months after a knife attack in Mannheim, Germany, in which five individuals were injured, and a police officer was killed in May 2024 [28]. The present study employed a cross-sectional observational design. In addition to a basic questionnaire assessing sociodemographic variables, three standardized instruments were administered. The PTSD Checklist for DSM-5 (PCL-5) assessed PTSS and PTSD [29], whereas the State–Trait Anger Expression Inventory–2 (STAXI-2) evaluated anger levels [30]. The German short form of the Childhood Trauma Questionnaire assessed experiences of childhood emotional neglect [31]. The sample included officers who were directly involved in the incident, and a control group of officers from the same department, who were not deployed due to different shift and working hours. Participation was voluntary, and no random sampling procedure was applied.

### 2.2. Participants

The final police report identified a total of 138 officers directly involved in the critical incident, of whom 78 (57%) voluntarily participated in the survey. Furthermore, 167 officers from the same police department served as a comparison group. Notably, 81 officers from this comparison group personally knew the deceased colleague, which enabled the formation of various subgroups depending on the tested hypotheses. Due to voluntary participation and the response rate among deployed officers, the possibility of selection bias cannot be excluded.

### 2.3. Ethical Approval

The study was approved by the Ethics Committee of Charité Berlin in accordance with the Declaration of Helsinki (EA4/085/18) and funded by the German Ministry of Defence (36K4–S322125). Participation was voluntary, and all participants provided written informed consent.

### 2.4. Data Collection

Six months after the incident and after approval by the department leadership, potential participants were informed about the study’s purpose. They received detailed explanations regarding voluntary participation, data protection through pseudonymization, the estimated time required to complete the questionnaire, and the potential for increased stress when responding. Support resources, both internal and external, were also provided. Questionnaires were subsequently distributed at specified collection points, prioritizing the deployed personnel, and were promptly retrieved thereafter.

### 2.5. Measurement Instruments and Statistical Evaluation

A basic questionnaire was used to collect socio-demographic variables, such as age, gender and years of service.

The Posttraumatic Stress Disorder Checklist (PCL-5) was used to assess PTSS [29]. This validated 20-item self-report measure aligns according to ICD-11 and DSM-5 criteria, covering symptom clusters of intrusion, avoidance, negative cognition, and hyperarousal. Items are rated on a 5-point Likert scale reflecting symptom intensity over the past month. A total score of 33 or higher is commonly used as a cut-off to indicate probable PTSD and the current research adopted this value. In the present sample, the PCL-5 demonstrated excellent internal consistency for the total score (Cronbach’s α = 0.919; 20 items). Subscale reliabilities were good to acceptable, with α = 0.822 for Intrusion (5 items), α = 0.821 for Avoidance (2 items), α = 0.804 for Negative Cognitions (7 items), and α = 0.757 for Arousal (6 items). The applicability of the PCL-5 cut-off to this specific police sample is based on prior validation studies; however, no population-specific validation was conducted within the present study.

Anger expression was assessed using the German version of the State–Trait Anger Expression Inventory-2 (STAXI-2), a 51-item self-report questionnaire rated on a 4-point Likert scale [30]. The inventory measures state, trait anger, and anger expression, including inward and outward expression as well as anger control. In the present study, trait anger is operationalized to a temporally stable disposition to perceive a wide range of situations as frustrating and to respond with elevated state anger. The STAXI-2 Trait Anger scale showed good internal consistency (α = 0.876; 10 items). For the subscales, internal consistency was α = 0.879 for Trait Anger Temperament (5 items) and α = 0.770 for Trait Anger Reaction (4 items).

To assess the impact of childhood emotional neglect on negative cognitions, participants completed the short form of the Childhood Trauma Questionnaire (CTQ) [30]. The German short form comprises 28 items, which are rated on a 5-point Likert scale. Childhood emotional neglect, assessed using the corresponding CTQ subscale, demonstrated acceptable internal consistency (α = 0.706; 5 items).

The statistical evaluation of the questionnaire was conducted using IBM SPSS Statistics for Windows (Version 21.0; IBM Corp., Armonk, NY, USA). To examine differences in sociodemographic variables between the deployed/non-deployed and acquainted/not-acquainted groups, chi-square (χ^2^) tests and independent-samples *t*-tests with α = 0.05 tests were conducted. Samples were examined for normal distribution before conducting *t*-tests. No violations were found, making the Mann–Whitney U test obsolete. To examine whether the deployed and non-deployed groups already differ significantly in their acquaintance status, a χ^2^-test was used. A χ^2^-test was also performed to assess differences in PTSD between the separate groups, as well as odds ratios (ORs) were calculated. Due to expected cell frequencies below 5, Fisher’s exact test was applied. To examine how PTSS affect the police officers who knew him compared to those who did not, a *t*-test was conducted, as well as to assess group differences in trait-anger. Homogeneity of variances was evaluated using Levene’s test; in case of violations, the degrees of freedom were adjusted downwards accordingly and indicated by a decimal place. Confidence intervals are reported rounded to one decimal place. To reduce the risk of Type I errors due to multiple comparisons the data were corrected using a family-wise error rate (FWER) correction with a significance threshold of *p* < 0.05. Additionally, a linear regression analysis was conducted to examine the effects of emotional neglect on negative cognitions in the context of PTSS. Data were examined for homoscedasticity, and when unequal variances were detected, Welch’s correction was applied to adjust the degrees of freedom. The statistical analyses were conducted to directly test the hypotheses formulated in the Introduction regarding deployment status, familiarity with the deceased, anger, and PTSS. Specifically, group comparisons were used to test Hypotheses 1 and 2, while regression analyses were conducted to examine Hypothesis 3.

## 3. Results

### 3.1. Demographics

No statistical differences were found in sociodemographic variables, such as gender, age (mean 37.2 years; standard deviation 10.7 years), or years of service between the groups categorized by deployment status or acquaintance with the deceased colleague (Table 1 and Table 2).

Five participants (2%) could not be classified due to missing information on the variables.

### 3.2. PTSD and PTSS

#### 3.2.1. PTSD and Deployment During the Incident

To assess the impact on PTSD of being deployed, a comparison was conducted between police officers who were deployed to the scene with their non-deployed counterparts by using the cut-off score. N = 245 police officers (n = 94 female; 39%) were included in the analysis. Among these, n = 78 (n = 24 female; 31%) were deployed, and n = 167 were not. Cross-tabulation indicated that 3% (5/167) of non-deployed officers met criteria for PTSD, compared to 6% (5/78) of deployed officers. This difference did not reach statistical significance.

#### 3.2.2. PTSD and Acquaintance with Killed Police Officer

To examine whether psychological symptoms were more pronounced among officers who were personally acquainted with the deceased, participants were categorized by that variable. In total, N = 241 police officers were included in the analysis. Among those not acquainted with the deceased officer, 0.8% (1/126) showed indications of PTSD, whereas 7.8% (9/115) of acquainted officers reached the clinical cut-off. A chi-square analysis revealed a significant association between knowing the fatally injured colleague and the presence of PTSD, χ^2^ (1, n = 241) = 7.48, *p* = 0.006, with an OR of 10.6 (95% CI: 1.3–85.1). Because some expected cell frequencies were below 5, Fisher’s exact test was conducted and confirmed statistical significance (*p* = 0.008).

#### 3.2.3. PTSS

To examine group differences in PCL-5 scores, independent *t*-tests were conducted. Although the group of officers who were deployed at the scene consistently showed higher mean scores across all PCL-5 subscales, none of these differences reached statistical significance. In contrast, the mean scores of officers who were acquainted with the deceased were significantly higher than those of officers who did not know him (Table 3).

### 3.3. Anger

The deployed police officers showed significantly higher mean scores on several subscales of the STAXI-2 questionnaire compared to those who were not deployed (Table 4). Specifically, the deployed officers reported greater frequency of anger experiences without specific provocation (Trait-Anger/Temperament) and increased anger in response to perceived frustration (Trait-Anger/Reaction).

In contrast, the two groups categorized according to their acquaintance with the deceased colleague differed significantly only on the Trait Anger Reaction subscale of the STAXI-2. Officers who were acquainted with the deceased showed significantly higher scores (T (239) = −1.8; *p* = 0.036; d = 0.23; 95% CI = [−0.5, −0.0]) compared to those who did not know him.

### 3.4. Influences of Childhood on PCL—Negative Cognitions

To examine the influence of predictor variables on the PCL-5 subscale Negative Cognitions, a linear regression analysis was conducted. The results indicated that acquaintance with the deceased and emotional neglect during childhood had statistically significant influences on the officers’ negative cognitions as a symptom of PTSD. While deployment status showed no significant effect, gender was marginally non-significant (*p* = 0.051) (Table 5).

## 4. Discussion

### 4.1. PTSD and PTSS

With respect to the first hypothesis, the present findings indicate that familiarity with the deceased colleague was associated with higher levels of PTSD and PTSS, whereas deployment status alone was not significantly associated with increased posttraumatic stress.

The present results indicate that deployment status alone does not account for the elevated risk of PTSS. Rather, acquaintance with the deceased emerged as a more substantial predictor among police officers. In this study, the prevalence of PTSD was 6.4% in the deployed group and 7.8% in the acquaintance group, which falls within the range reported for emergency responders in a systematic review by Wesemann et al. [32]. Although the prevalence of PTSD among deployed officers was approximately twice as high as that of non-deployed colleagues, the difference did not reach statistical significance, which may reflect sample-related limitations rather than a true absence of effects. In contrast, officers who were personally acquainted with the deceased colleague met the diagnostic criteria for PTSD significantly more often than those without such a personal connection. These findings are consistent with those reported by Pietrzak et al., who found that acquaintance with deceased was associated with a higher prevalence of PTSD [33]. Woller et al., suggested that police officers may experience heightened feelings of guilt and shame due to the nature of their profession, both of which are linked to an increased risk of developing PTSD [34]. Our analysis showed that police officers who knew the injured or deceased colleague had an approximately tenfold increased risk of having a PTSD (OR = 10.61; 95% CI [1.32–85.13]).

Moreover, the acquainted group exhibited higher PTSS scores across all PCL-5 subscales, suggesting a broader psychological impact. These findings align with previous research by Görlich et al., which identified similar associations between acquaintance and higher PTSS among firefighters [35]. Together, these results suggest that relational proximity to victims may serve as an independent risk factor for trauma-related distress, potentially amplifying emotional identification and cognitive processing of the event. From a psychological perspective, knowing the deceased may increase perceived personal relevance, empathy, and moral injury, thereby intensifying post-traumatic responses [36].

### 4.2. Anger

Regarding the second hypothesis, the results support the assumption that deployment to the incident scene is associated with higher levels of trait anger among police officers.

Although the acquaintance group showed higher levels of PTSS, anger expression as measured by the STAXI-2 was markedly higher among the deployed group [12]. The acquaintance group showed significant group differences only on the Anger Reaction subscale, indicating that officers who knew the deceased reported experiencing more intense anger in response to perceived criticism or negative evaluation by others.

Forbes et al. found that problematic anger among military populations increases with the frequency of exposure to critical incidents, yet it has received limited attention, indicating that it requires greater focus [37]. Consistent with this, the present study found that the deployed group exhibited significantly higher levels of trait-anger. As these findings stem primarily from military populations, differences in occupational roles, training, and operational contexts between military and police personnel limit direct comparability and warrant cautious interpretation when applied to police settings. In the present study, elevated trait anger among deployed officers represents an empirical finding, whereas associations between anger and PTSD symptom severity are derived from prior literature and were not directly tested. According to Spielberger et al. increased state anger is likely to reflect underlying chronic anger, particularly when both trait anger and inward-directed anger expression are elevated [14]. Future studies should examine this relationship more closely. This aligns with the hypotheses confirmed by Meffert et al., which suggest that higher trait anger predicts greater PTSD symptoms over time and that elevated PTSD symptoms at that time subsequently predict increased state anger [38]. Since the deployed group exhibited higher scores on the STAXI-2, the results of the study raise the question of whether this group might also show elevated levels of PTSD symptoms. However, these associations could not be confirmed in the present study. The relatively lower PCL-5 scores in the deployed group have been discussed in the literature in relation to various factors, such as differences in the operational roles of the deployed officers, access to institutional psychological support, individual resources, or a higher level of peer support, which has been associated with lower PTSS severity [39]. Similarly, Motreff et al. also identify these factors as a possible explanation discussed in prior research for the large variations in PTSD prevalence in their study [40].

### 4.3. Influences of Childhood on PCL—Negative Cognition

In line with the third hypothesis, childhood emotional neglect emerged as a significant predictor of negative trauma-related cognitions, alongside acquaintance with the deceased colleague.

McLean et al. demonstrated that negative cognitions mediate PTSD symptoms, highlighting their central role as a potential target for therapeutic intervention [41]. Accordingly, the present study investigates the impact of childhood emotional neglect on the development of negative cognitions. Our analysis indicated that emotional neglect had the most influential predictor in the model. Wesemann et al. emphasized that the role of emotional neglect in shaping negative cognitions related to the development of PTSD has thus far received limited empirical attention [32]. Our study found an association between the severity of negative cognitions and emotional neglect in childhood, consistent with previous findings demonstrating that a negatively shaped childhood can lead to enduring negative core beliefs, particularly in the context of perceived failure following trauma exposure [22,23,24,25,26]. Accordingly, the present findings demonstrate an association between childhood emotional neglect and negative trauma-related cognitions, while proposed developmental or cognitive mechanisms remain theoretical and were not directly examined in the present study. Future research should therefore prioritize examining this relationship to advance the understanding of underlying cognitive mechanisms. Our findings regarding the role of gender are consistent with the study by Wagner, which likewise found that gender had no significant influence on the development of PTSD, whereas incident-specific factors—such as personal acquaintance with the victim and being deployed at the scene—did show significant effects [27]. Although the literature on gender differences in PTSD remains inconsistent [42], we observed a trend toward significance in our data. It is possible that additional factors not assessed in the present study may moderate this association, such as social and occupational socialization, double burden of family and career and lower income [43].

Taken together, the present study contributes to the existing literature by simultaneously examining occupational exposure (deployment status), relational proximity to the deceased colleague, and developmental vulnerability (childhood emotional neglect) within a single cohort of police officers following one fatal duty-related incident. While previous studies have typically focused on these factors in isolation, the current findings suggest that relational proximity is more strongly associated with PTSS, whereas occupational exposure is primarily linked to anger-related outcomes. This integrated perspective helps to differentiate trauma-related symptom profiles in police populations and may inform more targeted post-incident support and intervention strategies.

## 5. Limitations

This study has several limitations that need to be considered. First, the representativeness of the sample, with a total of N = 245 participants, remains limited. Second, while the proportion of female participants corresponds to the actual gender distribution within the German police force [44], the relatively small sample size may have led to potential gender differences being overlooked.

The study relied on self-report questionnaires and voluntary participation, resulting in participation rate of 57% of deployed officers. As participation was voluntary and no random sampling procedure was applied, selection bias cannot be excluded. This may have influenced the accuracy of responses and led to potential selection bias. In addition, the measurement point—six months after the incident—without baseline data, weakens the interpretation of the results, as it remains unclear whether prior incidents may have affected the officers’ mental health. The cross-sectional design further precludes causal conclusions regarding the observed associations. Future studies should include regular monitoring to better track officers’ psychological well-being and to improve understanding of the effects of trauma exposure.

## 6. Conclusions

This study examined posttraumatic stress symptoms, anger, and negative trauma-related cognitions among police officers following a fatal duty-related incident, with a particular focus on deployment status, familiarity with the deceased colleague, and childhood emotional neglect.

The findings indicate that familiarity with the deceased colleague was more strongly associated with PTSD and PTSS than deployment to the incident scene, whereas deployment status was primarily related to elevated levels of trait anger. In addition, childhood emotional neglect emerged as a significant predictor of negative trauma-related cognitions, highlighting the relevance of developmental vulnerability in the context of occupational trauma. The novel contribution of this study lies in the simultaneous examination of relational, occupational, and developmental risk pathways within a single cohort of police officers who were exposed to the same fatal incident. By considering these factors jointly, the present findings extend previous research that has predominantly investigated these dimensions in isolation.

From a practical perspective, the results underscore the importance of post-incident support strategies that account not only for direct operational exposure but also for relational proximity to affected colleagues and individual vulnerability factors. In particular, interventions addressing anger regulation and trauma-related cognitions may be beneficial for specific subgroups of police officers. Given the cross-sectional design and reliance on self-report measures, the conclusions should be interpreted with caution. Future research should employ longitudinal designs and repeated assessments to further clarify the temporal dynamics of PTSS, anger, and cognitive responses following critical incidents in police populations.

## Figures and Tables

**Table 1:** Differences in gender, age, and years of service between deployed and non-deployed officers.

Variable	Age	Years of Service	Gender	
			Female	Male
Deployed group (Mean ± SD) or (n (%))	36.9 ± 10.4	14.0 ± 10.9	n = 24 (10%)	n = 52 (21%)
Non-deployed group (Mean ± SD) or (n (%))	37.5 ± 10.9	14.4 ± 11.1	n = 70 (29%)	n = 98 (40%)
Test statistic	T (236) = 0.3	T (241) = 0.3	*χ*^2^_(1,*n* = 244)_ = 2.2	
*p* Value	0.733	0.781	0.134	
Acquainted group (Mean ± SD) or (n (%))	38.4 ± 10.4	15.5 ± 10.8	n = 47 (20%)	n = 70 (29%)
Not-acquainted group (Mean ± SD) or (n (%))	36.4 ± 11.0	13.2 ± 11.1	n = 44 (18%)	n = 79 (33%)
Test statistic	T (232) = −1.4	T (237) = −1.7	*χ*^2^_(1,*n* = 240)_ = 0.5	
*p* Value	0.157	0.100	0.483	

SD = standard deviation; T = *t*-test; *χ*^2^ = *χ*^2^-test.

**Table 2:** Grouping of participants by deployment status and acquaintance with the deceased.

Variable	Not Acquainted with the Deceased (n (%))	Acquainted with the Deceased (n (%))	Test Statistic	*p* Value
Non-deployed group	n = 83 (34%)	n = 81 (33%)	*χ*^2^_(1,*n* = 241)_ = 0.58	0.448
Deployed group	n = 43 (17%)	n = 34 (14%)		
Total	n = 126 (51%)	n = 115 (47%)		

SD = standard deviation; *χ*^2^ = *χ*^2^-test.

**Table 3:** Independent *t*-tests for differences in PCL-5 scores between officers acquainted and not acquainted with the deceased.

Variable	N Acquainted (Not Acquainted)	Mean (SD) Acquainted	Mean (SD) Not Acquainted	df	t-Value	*p* Value	Cohen’s *d*	95% CI	
								Lower	Upper
Intrusions	115 (126)	3.61 (3.34)	2.40 (2.58)	214.2	−3.1	0.010 *	0.41	−2.0	−0.4
Avoidance	115 (126)	1.60 (1.97)	0.78 (1.38)	201.8	−3.7	<0.001 *	0.49	−1.3	−0.4
Negative Cognitions	115 (126)	3.03 (4.09)	1.71 (2.49)	185.1	−2.9	0.002 *	0.39	−2.2	−0.4
Arousal	115 (126)	3.33 (3.55)	2.40 (2.68)	211.3	−2.3	0.012 *	0.30	−1.7	−0.1
Total-Score	115 (126)	11.57 (11.54)	7.31 (7.40)	191.2	−3.4	<0.001 *	0.44	−6.7	−1.8

CI = Confidence Interval; df = degrees of freedom (with decimal due to lack of homoscedasticity); PCL-5 = Post-traumatic Stress Disorder Checklist; SD = standard deviation. * After correcting for the family-wise error rate, the result is significant at the *p* < 0.05 level.

**Table 4:** Independent *t*-tests for differences in STAXI-2 trait-anger scores between deployed and non-deployed officers.

Variable	N Deployed (Not Deployed)	Mean (SD) Deployed	Mean (SD) Not Deployed	df	t-Value	*p* Value	Cohen’s *d*	95% CI	
								Lower	Upper
Trait-Anger	78 (167)	19.38 (5.25)	17.81 (4.82)	243	2.3	0.011	0.32	0.2	2.9
Trait-Anger/ Reaction	78 (167)	9.40 (2.63)	8.72 (2.48)	243	1.9	0.028	0.26	0.0	1.4
Trait-Anger/ Temperament	78 (167)	8.46 (2.94)	7.78 (2.61)	243	1.8	0.034	0.25	−0.1	1.4

CI = Confidence Interval; df = degrees of freedom; SD = standard deviation; STAXI-2 = State–Trait Anxiety Inventory-2.

**Table 5:** Linear regression analysis testing the influence of emotional neglect, acquaintance, deployment, and gender on the dependent variable negative cognition.

Predictor	B	Std. Error	*β*	*t*-Value	*p* Value
Emotional Neglect	0.23	0.069	0.20	3.3	0.001
Deployment	0.28	0.455	0.04	0.6	0.536
Acquaintance	0.40	0.141	0.18	2.9	0.005
Gender	0.86	0.441	0.12	2.0	0.051
R^2^	0.090				
F (4,236) = 5.827					<0.001

B = unstandardized coefficient; Std. Error = standard error; *β* = standardized coefficient.

## Data Availability

The data collected for this study is not publicly available due to privacy and ethical restrictions. Limited insight may be granted after correspondence with the author and permission of the Bundeswehr Hospital Berlin.
